# Strain‐Modulated Reconfigurable Optical Information Processing in Flexible Graphene/PDMS

**DOI:** 10.1002/advs.75955

**Published:** 2026-06-03

**Authors:** Zexin Cui, Lihua Tong, Yuehua Wang, Mengting Jiang, Jiaxu Sun, Huabo Song, Qifan Li, Guangyuan Cui, Churui Guo, Wentao Meng, Shaoya Wang, Yuee Chen, Yanling Wu, Xiaodan Xu

**Affiliations:** ^1^ State Key Laboratory of Metastable Materials Science and Technology & Hebei Key Laboratory of Microstructural Material Physics School of Science Yanshan University Qinhuangdao China; ^2^ Beijing Key Laboratory of Nanophotonics and Ultrafine Optoelectronic Systems Key Laboratory of Advanced Optoelectronic Quantum Architecture and Measurements of Ministry of Education School of Physics Beijing Institute of Technology Beijing China

**Keywords:** graphene, optical logic gate, optical switching, spatial self‐phase modulation, strain engineering

## Abstract

All‐optical information processing, featuring ultrafast response and immunity to electromagnetic interference, plays a pivotal role in future communications and computing technologies. Graphene, with its exceptional nonlinear optical properties and mechanical flexibility, emerges as an ideal candidate for flexible photonic devices. However, graphene‐based photonic components are typically functionally fixed and lack dynamic reconfigurability. Here, we integrate graphene with polydimethylsiloxane (PDMS) to fabricate a flexible graphene/PDMS composite and investigate its spatial self‐phase modulation (SSPM) effect under mechanical strain. Our results demonstrate that the nonlinear optical response of the composite can be dynamically tuned by strain. Under 532 nm laser excitation (intensity 35 W/cm^2^), increasing the tensile strain from 0% to 40% continuously suppresses the number of SSPM diffraction rings from 8 to 0, while accompanied by a reduction in the third‐order nonlinear susceptibility $\chi_{\mathrm{monolayer}}^{\left(3\right)}$ from 1.357 × 10^−7^ to 6.125 × 10^−8^ e.s.u. This tunable SSPM effect originates from strain‐induced modifications in both the effective number of optically interacting layers and the electronic band structure of graphene. Leveraging this mechanism, we further designed a strain‐gated optical switch and reconfigurable optical logic gates, enabling flexible switching between “OR” and “AND” gates. This work opens new avenues for graphene‐based tunable nonlinear photonic devices.

## Graphical abstract

Graphene/PDMS—a stable, mechanically robust, and highly tunable flexible composite, exhibits excellent strain‐tunability. The spatial self‐phase modulation (SSPM) effect can be reversibly and continuously modulated by strain. A strain‐gated optical switch can be constructed, thereby realizing reconfigurable optical logic gates and reversible switching between “OR” and “AND” gates.

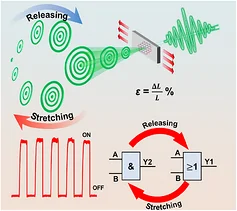

## 1 Introduction

All‐optical information processing has emerged as a pivotal direction for future information technologies, owing to its unparalleled advantages in speed, bandwidth, parallelism, and immunity to electromagnetic interference [1]. Spatial self‐phase modulation (SSPM), arising from the optical Kerr effect, represents a powerful approach for optical field control and all‐optical information processing [2]. SSPM has been widely employed to characterize the third‐order nonlinear optical response of various two‐dimensional (2D) materials [3, 4] and has enabled the development of diverse nonlinear photonic devices. In 2015, Wu et al. first proposed an all‐optical switch based on SSPM, achieving dual‐wavelength mutual control of the SSPM diffraction rings [5]. Subsequently, SSPM‐based photonic diodes [6], modulators [7], and logic gates [8, 9] were successively developed, enabling information control over light intensity, phase, and spatial modes. Building upon the all‐optical switch, Xu et al. manipulated SSPM diffraction ring patterns by tailoring the focal position and intensity of control beams, thereby realizing nine fundamental Boolean functions and accelerating the development of all‐optical processors [10]. Although SSPM has demonstrated great potential in constructing nonlinear photonic devices, numerous studies on SSPM of 2D materials are conducted relying on N‐methyl‐2‐pyrrolidone (NMP) dispersions [11, 12, 13], which exhibit poor stability and controllability—major obstacles for practical device integration [14, 15]. Several strategies have been explored to address these limitations, such as vertical illumination [16], embedding in polymer matrices (e.g., PMMA) [6], and increasing dispersion viscosity (e.g., by adding agar) [17]. Moreover, conventional SSPM‐based photonic devices are typically functionally fixed, lacking the capability for dynamic reconfiguration—a critical bottleneck that severely restricts their versatility and broad adoption.

Graphene possesses exceptionally high carrier mobility and excellent field‐effect switching ratios, making it an ideal material for constructing field‐effect transistors (FETs) and digital logic devices [18, 19]. Its tunable band structure enables efficient photon absorption, emission, and photoelectric conversion, underpinning remarkable performance in optoelectronic applications [20, 21, 22]. In nonlinear optics, graphene also exhibits remarkable properties that warrant attention. Specifically, its strong third‐order nonlinear optical responses, such as high‐harmonic generation and four‐wave mixing, are essential for all‐optical signal processing, wavelength conversion, and quantum information processing [23]. Moreover, graphene demonstrates superior SSPM response, with its third‐order nonlinear susceptibility ($\chi_{\mathrm{monolayer}}^{\left(3\right)}$ reaching up to 10^−7^ e.s.u.) surpassing that of many other 2D counterparts [24, 25]. Owji et al. fabricated polyurethane‐graphene (PU‐G) composites and observed enhanced SSPM responses, demonstrating the potential of graphene‐based composite systems for nonlinear photonics [26]. Recent studies have confirmed the feasibility of employing graphene in nonlinear femtosecond all‐optical switches [27] and reconfigurable nanophotonic devices [28].

Strain engineering, as an efficient modulation technology, can tailor the physical properties of materials by modifying their crystal lattice and electronic structures, thereby influencing electrical, optical, and magnetic behaviors [29, 30, 31, 32]. Integrating 2D materials with flexible substrates enables mechanical strain to induce piezoresistive [33] and piezoelectric effects [34, 35], expanding their applications in flexible sensors, optoelectronics, and wearable technologies. Graphene, in particular, exhibits extraordinary ductility and flexibility, sustaining tensile strains up to 25% without fracturing [36]. Yu et al. successfully applied uniaxial strain to monolayer graphene on a flexible polydimethylsiloxane (PDMS) substrate. They discovered that graphene resistance increased linearly when uniaxial tensile strain exceeded 2.47% [37], leading to the development of high‐performance strain sensors. More recent studies have shown that strain engineering can break graphene's sublattice symmetry to induce second‐order nonlinear optical responses [38] and even enable high‐harmonic generation (HHG) with high dynamic range [39]. This strain–optics coupling mechanism opens new pathways for reconfigurable all‐optical devices. For the SSPM investigation, the sample systems are collections of flakes spatially dispersed in liquid or gel, which pose a significant challenge for strain modulation. Therefore, the strain‐tunable SSPM effect in graphene remains largely unexplored. Whether mechanical strain can be harnessed to actively control SSPM and thereby enable novel classes of nonlinear optical devices remains an open and compelling question.

In this work, we construct a stable, mechanically robust, and highly tunable flexible composite (graphene/PDMS) by integrating graphene onto a PDMS substrate. In SSPM experiments, this platform eliminates the common issue of diffraction ring collapse observed in liquid dispersions. Crucially, the graphene/PDMS composite exhibits excellent strain‐tunability, with its SSPM response being reversibly and continuously modulated by strain. Leveraging this strain‐tunable nonlinearity, we demonstrate a strain‐gated optical switch and reconfigurable optical logic gates capable of reversible switching between the “OR” and “AND” functions. Our findings establish a foundational framework for advancing graphene‐based passive nonlinear photonic devices with on‐demand reconfigurability.

## 2 Sample Preparation and Characterization

**FIGURE 1:**
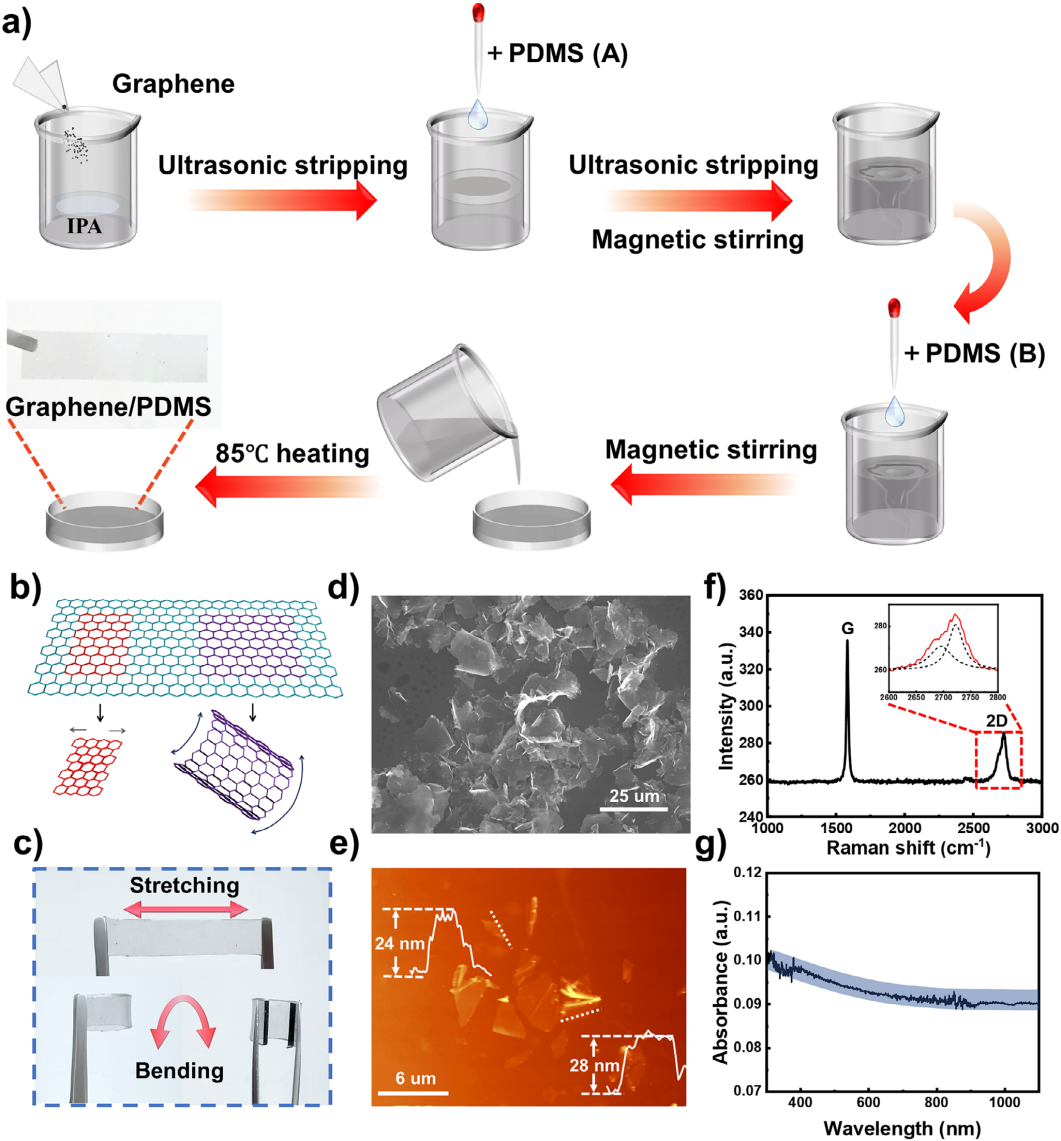
Preparation process and characterization of graphene/PDMS composite. (a) Schematic of sample preparation procedure. (b) Graphene's crystal structure and flexibility. (c) Demonstration of the physical flexibility of graphene/PDMS composite. (d, e) Scanning electron microscopy (SEM) and atomic force microscopy (AFM) images of graphene. (f, g) Raman spectrum and absorption spectrum of the graphene/PDMS composite.

PDMS—polydimethylsiloxane, with repeating units (C_2_H_6_OSi)_n_, is a high‐molecular‐weight organosilicon polymer composed of a base elastomer (PDMS A) and a curing agent (PDMS B). It possesses high viscosity and excellent elasticity [40] and is widely employed as a substrate for transferring 2D materials [41]. Graphene, composed of carbon atoms arranged in a hexagonal honeycomb lattice through sp^2^ hybridization, exhibits exceptional mechanical flexibility, enabling significant bending and stretching, as illustrated in Figure 1b [42]. In this work, graphene nanosheets were uniformly dispersed within PDMS to fabricate a flexible graphene/PDMS composite. The preparation procedure is shown in Figure 1a. First, 1 mg of graphene was added to 10 mg of isopropanol (IPA) and sonicated for 2 h to weaken interlayer van der Waals forces, thereby obtaining few‐layer graphene flakes [43]. Subsequently, 9 g of PDMS base (A) was added, followed by 2 h of ultrasonic treatment and 2 h of magnetic stirring to ensure homogeneous dispersion of graphene. Then, 1 g of curing agent (B) was added, and the mixture was stirred magnetically for an additional 2 h to achieve uniform blending. After standing at room temperature for 1.5 h to allow entrapped bubbles to dissipate, the mixture was heated at 85°C for 50 min to promote crosslinking and further eliminate residual voids, ultimately yielding a flexible graphene/PDMS composite. The resulting sample has a thickness of approximately 1.1 mm, appears transparent with a light gray tint, and exhibits exceptional flexibility—capable of being freely bent and stretched, as shown in Figure 1c.

The graphene materials used in this study were commercially sourced from Nanjing XFNANO Materials Technology Co., Ltd. Scanning electron microscopy (SEM) imaging (Figure 1d) shows graphene flakes with typical lateral dimensions of approximately 10 µm and surfaces free of visible contaminants [44]. Atomic force microscopy (AFM) measurements (Figure 1e) reveal that the thickness of these nanosheets is around 20–30 nm, which remains consistent in all measurements. Raman spectroscopy (excitation wavelength: 532 nm) further confirms the high quality of the graphene material, as shown in Figure 1f. The characteristic G peak appears at 1582 cm^−1^, corresponding to in‐plane vibrations of sp^2^‐bonded carbon atoms, while the 2D peak near 2720 cm^−1^ arises from a double‐resonant two‐phonon scattering process [45]. Upon transitioning from monolayer to multilayer configurations, graphene exhibits modified interlayer interactions that significantly perturb its electronic band structure. This perturbation introduces additional scattering channels, resulting in the splitting of the characteristic 2D Raman peak into two distinct sub‐peaks. Notably, the absence of a discernible D peak (∼ 1350 cm^−1^) indicates a low defect density and high crystalline quality. The ultraviolet–visible–near infrared (UV–vis–NIR) absorption spectrum of the graphene/PDMS composite (Figure 1g) exhibits broadband absorption across the 300–1100 nm range, highlighting its potential for broadband photonic applications.

## 3 Strain‐Modulated SSPM Effect

**FIGURE 2:**
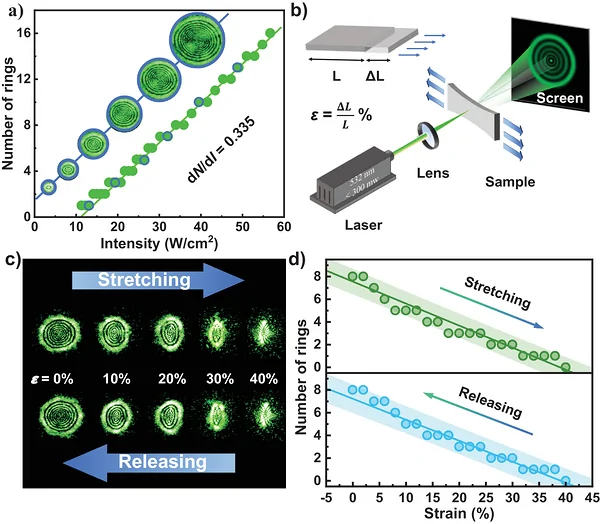
SSPM diffraction rings of the graphene/PDMS composite under varying strain. (a) Evolution of diffraction rings with incident light intensity. (b) Schematic of the strain‐applying apparatus. (c) Variation of the diffraction ring with the stretching and releasing of strain (0%–40%). d) Dependence of diffraction ring number on applied strain.

SSPM experiments were conducted on the graphene/PDMS composite at a wavelength of 532 nm, and the representative results are shown in Figure 2a. The results show that when the laser intensity exceeds a certain threshold, concentric diffraction rings emerge, and the number of rings (*N*) increases linearly with incident light intensity (*I*), with a slope d*N*/d*I* = 0.335. This linear dependence indirectly reflects the material's third‐order nonlinear optical properties. Based on Equations (1) and (2), we quantitatively determine the nonlinear refractive index (*n*_2_) and the third‐order nonlinear susceptibility ($\chi_{\mathrm{monolayer}}^{\left(3\right)}$) [46]:

(1) $$n_{2}=\frac{\lambda }{2n_{0}L_{\mathrm{eff}\;}}\;\cdot \frac{N}{I}$$

(2) $$\chi_{\mathrm{monolayer}}^{\left(3\right)}=\frac{c\lambda n_{0}}{2.4\times 10^{4}\pi^{2}L_{\mathrm{eff}\;}N_{\mathrm{eff}}^{2}}\;\cdot \frac{dN}{dI}$$

Here, the laser wavelength is *λ* = 532 nm, the linear refractive index of PDMS is *n*_0_ = 1.41, and the effective optical thickness of the sample is *L*_eff_  = 1.1 mm. *N*_eff_ represents the effective number of graphene layers interacting with the laser beam. Based on the graphene concentration [47], *N*_eff_ is estimated to be 146 (details see Section S1). From these parameters, we obtain *n*_2_ = 5.7 × 10^−5^  cm^2^/W and $\chi_{\mathrm{monolayer}}^{\left(3\right)}$ = 1.357 × 10^−7^ e.s.u., which is consistent with the order of magnitude reported for graphene dispersions (*n*_2_ = 2.5 × 10^−5^ cm^2^/W, $\chi_{\mathrm{monolayer}}^{\left(3\right)}$ = 1 × 10^−7^ e.s.u.) [3]. Notably, unlike liquid dispersions, the graphene/PDMS composite exhibits no asymmetric or collapsed ring structures, indicating that PDMS serves as an excellent inert host capable of preserving graphene's intrinsic third‐order nonlinearity. SSPM measurements conducted at random locations across the sample yield identical ring patterns and counts (see Figure S1), confirming excellent spatial homogeneity of the prepared sample. As a control, pure PDMS films of identical thickness show no SSPM response under the same conditions (Figure S2).

Owing to its excellent flexibility, the graphene/PDMS composite can be subjected to uniaxial stretching and releasing at will. We integrated a tensile stage into the SSPM setup (Figure 2b) to investigate strain‐dependent nonlinear optical behavior. Strain value *ε* is defined as [48]:

(3) $$\varepsilon =\frac{\Delta L}{L}\;.100\%$$

where ∆*L* is the elongation, and *L* is the original gauge length (*L* = 10 mm). The translation stage was advanced in 0.2 mm increments, corresponding to an *ε* value of 2% per step. Remarkably, the number of diffraction rings (*N*) decreases monotonically with increasing strain and fully recovers with strain release, demonstrating reversible tunability. Figure 2c shows the evolution of SSPM diffraction rings during stretching and releasing processes. Moreover, during the stretching process, the PDMS morphology only underwent uniform thinning, with almost no other types of deformation, which eliminates the potential interference with the SSPM diffraction rings (Section S4). Figure 2d presents the number of diffraction rings as a function of strain value. As the *ε* increases from 0% to 40%, the ring number decreases from 8 to 0; with the *ε* releasing back to 0%, it fully recovers to 8, confirming excellent reversibility.

**FIGURE 3:**
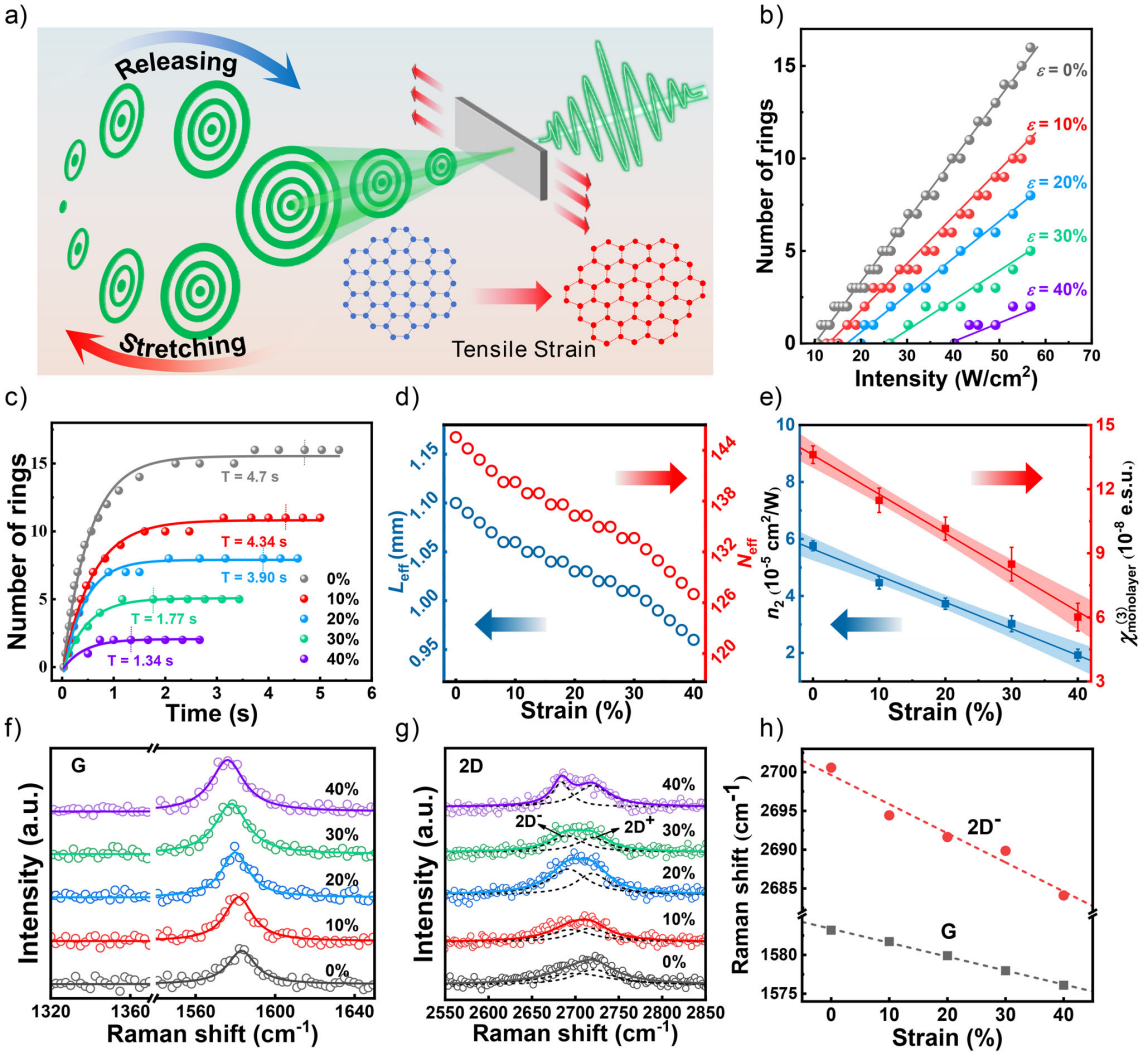
Strain‐modulated SSPM effect. (a) Reversible tuning of SSPM diffraction rings by strain. (b) Dependence of diffraction ring number on laser intensity at different strain levels (0%, 10%, 20%, 30%, and 40%). (c) Dependence of diffraction ring formation time on strain level (0%, 10%, 20%, 30%, and 40%) at a fixed laser intensity of 56.72 W/cm^2^. (d) Variation of sample effective thickness (*L*_eff​_) and corresponding number of graphene layers (*N*_eff_) with strain. (e) Strain‐dependent nonlinear refractive index *n*_2​_ and monolayer third‐order nonlinear susceptibility $\chi_{\mathrm{monolayer}}^{\left(3\right)}$. Error bars denote standard deviations from three independent measurements, while the shaded region indicates the 95% confidence interval of the linear regression. (f, g) Spectral evolution of the G and 2D Raman peaks as a function of applied strain, illustrating their shift sensitivity (h).

In the reversible strain‐controlled SSPM experiment (Figure 3a), we further investigated the strain‐modulation characteristics of the third‐order nonlinear optical coefficients in graphene/PDMS. Intensity‐dependent SSPM measurements were conducted at different tensile strain levels (0%, 10%, 20%, 30%, and 40%), and the number of diffraction rings (*N*) was recorded as a function of incident laser intensity (*I*). In Figure 3b, it can be observed that the slope d*N*/d*I* gradually decreases with increasing strain, while the switching threshold intensity progressively increases. Additionally, we measured the time required for the diffraction rings to reach a stable state at different strain levels under a fixed laser intensity of 56.72 W/cm^2^. Figure 3c shows that after a few seconds of interaction, the diffraction ring number reaches its maximum. This process can be described according to the “wind‐chime” model [5] (details see Section S5). In addition, the formation time of the diffraction rings progressively shortens with increasing strain, indicating that strain can effectively modulate the response time of the SSPM effect.

According to Equations (1) and (2), we extracted *n*_2_ and $\chi_{\mathrm{monolayer}}^{\left(3\right)}$ at each strain level as summarized in Table 1. The variation trend of *n*_2_ and $\chi_{\mathrm{monolayer}}^{\left(3\right)}$ values with strain modulation is shown in Figure 3e. As the strain increases from 0% to 40%, *n*_2_ decreases from 5.743 × 10^−5^ to 1.962 × 10^−5^ cm^2^/W. This is partly attributed to the reduction in the effective optical thickness *L*_eff_ of the sample with increasing tensile strain, which concurrently leads to a decrease in the effective number of layers interacting with light *N*_eff_. The dependence of both *L*_eff_ and *N*_eff_ on applied strain is shown in Figure 3d. More fundamentally, mechanical strain is transferred from the PDMS matrix to the embedded graphene flakes, inducing lattice distortion that alters graphene's electronic band structure, symmetry, and polarizability, thereby modulating its intrinsic nonlinear optical properties [49]. To understand the strain dependence of the electronic structure of graphene, we carried out electronic band structure calculations of graphene under various types of strain (Section S6). A bandgap opening of various strained graphene at the K point of the Brillouin zone is clearly seen, which can be attributed to the breaking of sublattice symmetry [50]. Conceivably, $\chi_{\mathrm{monolayer}}^{\left(3\right)}$ decreases from 1.357 × 10^−7^ to 6.125 × 10^−8^ e.s.u. (Figure 3e), directly reflecting strain‐modified graphene's crystal and electronic structure. As a control group, we prepared graphene/PDMS samples with varying thicknesses (0.95–1.19 mm) and measured their *n*_2_ under 0% strain (Figure S6). The sample thickness variation associated with 40% strain (from 1.10 to 0.96 mm) can induce an *n*_2_ variation of 14% (from 5.70 × 10^−5^ to 4.91 × 10^−5^ cm^2^/W), which is smaller than the reduction in *n*_2_ induced by strain (5.743 × 10^−5^ to 1.962 × 10^−5^ cm^2^/W). It is confirming that thickness parameter alone cannot account for the dramatic nonlinear coefficient changes observed. Therefore, the dominant mechanism underlying the tunable third‐order nonlinearity is the strain‐driven modification of graphene's intrinsic electronic structure, with secondary contributions from *N*_eff_.

**TABLE 1:** Strain‐dependent d*N*/d*I*, *n*_2_, and *χ* ^(3)^ of the graphene/PDMS composite.

ɛ (%)	d*N*/d*I* (cm^2^/W)	*n*_2_ (cm^2^/W)	$\chi_{\mathrm{total}}^{\left(3\right)}\left(e.s.u\right)$	$\chi_{\mathrm{monolayer}}^{\left(3\right)}\left(e.s.u\right)$
0	0.335	5.743 × 10^−5^	2.892 × 10^−3^	1.357 × 10^−7^
10	0.251	4.463 × 10^−5^	2.248 × 10^−3^	1.147 × 10^−7^
20	0.201	3.677 × 10^−5^	1.852 × 10^−3^	1.001 × 10^−7^
30	0.163	3.054 × 10^−5^	1.538 × 10^−3^	8.564 × 10^−8^
40	0.100	1.962 × 10^−5^	9.879 × 10^−4^	6.125 × 10^−8^

To experimentally verify that the embedded graphene flakes indeed experience measurable strain and to characterize the resulting structural modifications, we conducted Raman spectroscopy measurements under varying tensile strains (0%, 10%, 20%, 30%, 40%). The acquired spectra were fitted using Lorentzian lineshapes to track peak evolution. Figure 3f,g shows the spectral evolution of the G and 2D peaks as a function of applied strain. As the graphene samples are multilayer flakes, the 2D peak splits into two main subpeaks (i.e., 2D^−^ and 2D^+^), referring to the polarizations parallel and perpendicular to the applied strain [51]. As shown in Figure 3h, increasing the tensile strain from 0% to 40% induces a redshift of 7.09 cm^−1^ in the G peak and a significantly larger redshift of 16.48 cm^−1^ in the 2D^−^ peak—more than twice the shift rate for the G peak. The higher shift sensitivity of the 2D^−^ peak arises from the combined effects of phonon dispersion and electronic band structure evolution [52]. Furthermore, we can quantify the strain transfer efficiency by using the Gruneisen parameter (*γ* = ‐∂ω/(∂ε∙ω_0_)) [53]. For the multilayer graphene system in this literature, the 2D peak shift rate (∂ω/∂ε) is approximately −8 cm^−^^1^/%, corresponding to a Grüneisen parameter of −0.30. Based on this, we calculate that the 40% macroscopic stretching of the sample results in an effective strain of approximately 2.0% within the graphene crystal lattice and a strain transfer efficiency of 5.0%. However, due to differences in the strain application direction and the actual number of layers, the real transfer efficiency in our experiment may vary slightly. This strain effectively induces a bandgap opening that suppresses electron transitions from the valence to the conduction band, thereby affecting the excitation of the bandgap‐dependent SSPM phenomenon. In contrast, the 2D^+^ subpeak exhibits negligible shift under strain, reflecting the anisotropic response of the electronic and phonon bands to uniaxial deformation. In this system, graphene is fully embedded in PDMS, which provides enhanced protection and prevents potential delamination caused by tensile strain. Moreover, in the strain‐dependent Raman measurements (Figure 3f), no defect peak (D peak at 1350 cm^−1^) appeared. Therefore, we believe that virtually no delamination or structural defects occur during the stretching process.

## 4 Strain‐Modulated Optical Switches and Reconfigurable Logic Gates

**FIGURE 4:**
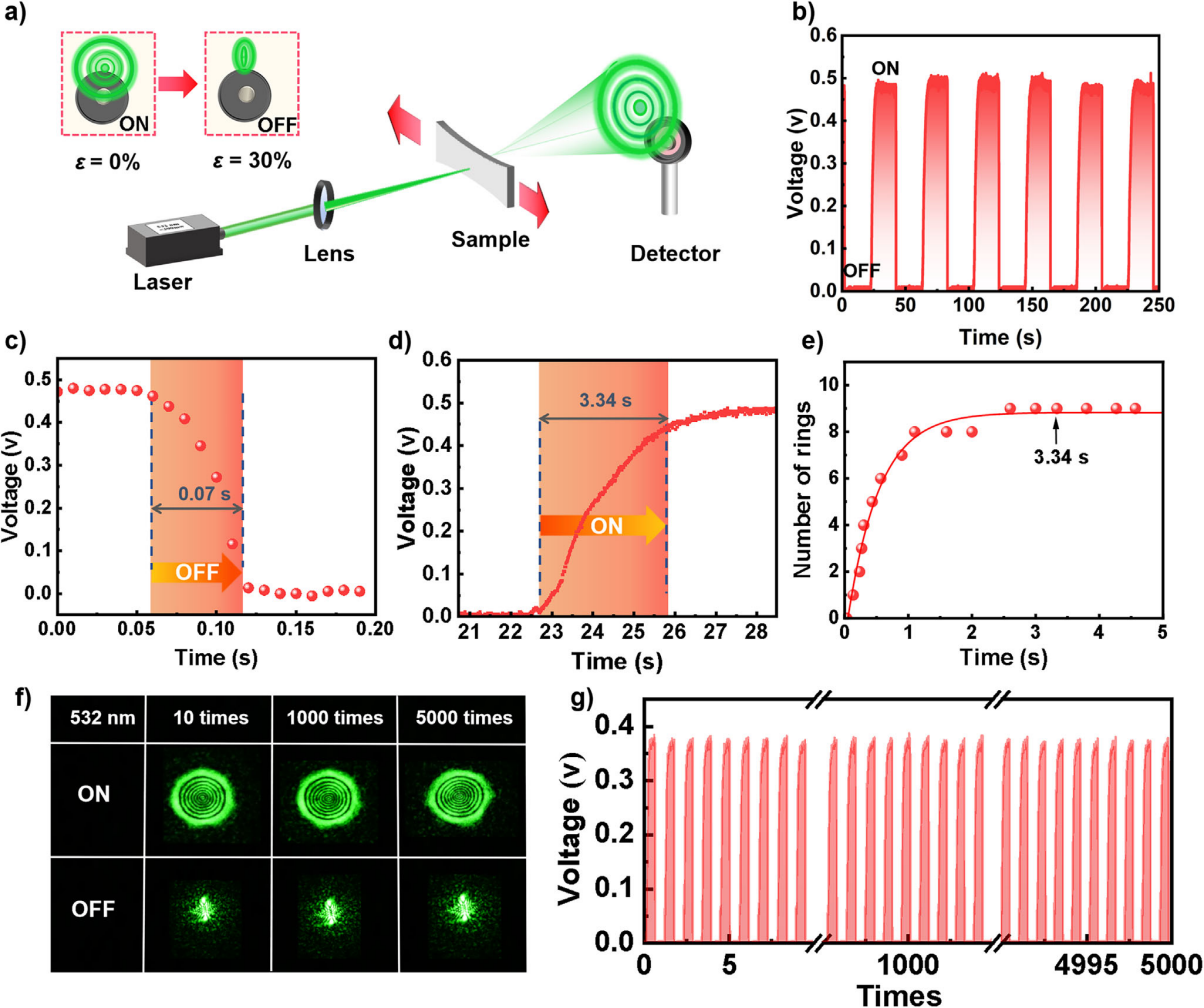
Strain‐tunable optical switching. (a) Experimental setup for the optical switch at 532 nm. (b) Periodic optical switching signal. (c) Falling edge corresponding to the “OFF” signal. (d) Rising edge corresponding to the “ON” signal. (e) Temporal evolution of SSPM diffraction rings. (f, g) The stretching‐releasing cycle stability tests on the graphene/PDMS sample.

We demonstrate a strain‐modulated optical switch by cyclically stretching the graphene/PDMS composite to *ε* = 30% and releasing it back to *ε* = 0% using a motorized translation stage, as illustrated in Figure 4a. The laser intensity is fixed at 37.81 W/cm^2^, and the outermost SSPM diffraction ring is monitored by a photodetector, yielding a periodic output signal. In the unstrained state (*ε* = 0%), the detector registers the outermost ring, corresponding to the “ON” state; after applying strain (*ε* = 30%), the number of rings decreases such that the outermost ring disappears, resulting in an “OFF” state (Figure 4b). Interestingly, the duration of signal “ON” and “OFF” exhibits a pronounced asymmetry. The translation stage operates at a speed of 23.7 mm/s, requiring 0.12 s to reach 30% strain. Figure 4c records the falling edge of the switching signal transitioning from “ON” to “OFF” occurring within approximately 0.07 s. This rapid response is due to the fact that the diffraction ring count begins to diminish before reaching the full 30% strain. In contrast, the rising edge (from “OFF” back to “ON”) takes a significantly longer time (∼ 3.34 s), as shown in Figure 4d. This asymmetry stems from the finite time required for the SSPM pattern to re‐establish after strain release. Consistently, Figure 4e tracks the temporal evolution of ring formation, revealing that the diffraction pattern recovers on a timescale consistent with the rising edge duration. Such asymmetric switching dynamics could be advantageously exploited in tailored pulse shaping or precise temporal gating [54, 55]. Moreover, we conducted multiple tensile‐release cycle tests on the graphene/PDMS sample, as shown in Figure 4f,g. The results show that after 5000 cycles of stretching‐releasing tests, the SSPM effect could be stably modulated, and the optical switch remained available, indicating good durability and stability of the system.

**FIGURE 5:**
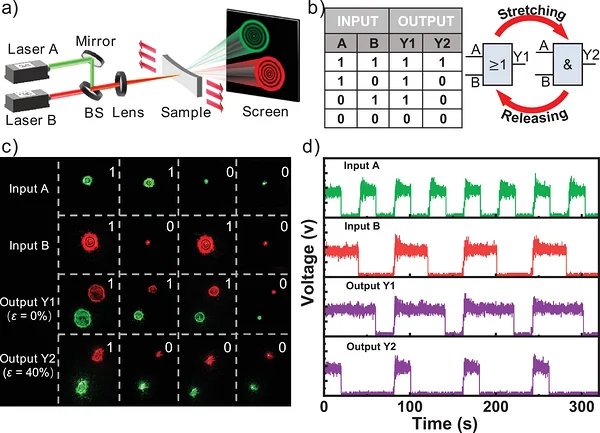
Strain‐modulated reconfigurable logic gates based on graphene/PDMS. (a) Experimental setup for a strain‐reconfigurable optical switch. (b) Signal transmission states and equivalent switching circuits for “OR” and “AND” logic operations. (c) Strain‐modulated reconfiguration between “OR” and “AND” logic gates. (d) Temporal output signals corresponding to the logic operations in c).

The SSPM effect further enables the implementation of various optical logic operations for optical signal processing. Figure 5a presents the experimental configuration for a strain‐reconfigurable optical logic gate, where 532 and 671 nm laser beams are alternately selected as control light and signal light. Figure 5b illustrates the signal transmission states of the “OR” and “AND” logic gates along with their corresponding switch circuit configurations. The combination of inputs A and B with output Y1 implements an “OR” gate, while A and B, with output Y2, form an “AND” gate. Critically, by introducing mechanical strain as a reconfiguration parameter, the device can be reversibly switched between these two logic functions. In our demonstration (Figure 5c), the logical states are encoded based on the presence (“1”) or absence (“0”) of far‐field diffraction rings. The switching thresholds are set at intensities $I_{532}^{1}$ = 33.02 W/cm^2^, $I_{532}^{0}$ = 8.62 W/cm^2^ for the 532 nm beam and $I_{671}^{1}$ = 27.80 W/cm^2^, $I_{671}^{0}$ = 10.78 W/cm^2^ for the 671 nm beam. In the absence of strain, the system operates as an “OR” gate; at 40% tensile strain, it transforms into an “AND” gate—a fully reversible transformation. By manipulating the timing of the input optical signals, it is possible to dynamically alternate between the two logic outputs over time, as shown in Figure 5d, where high (“1”) and low (“0”) logic levels correspond respectively to the presence or absence of diffraction rings. This strain‐mediated reconfigurability underscores the potential of graphene/PDMS as a versatile platform for adaptive, optical information processing, where mechanical degrees of freedom provide an additional dimension for real‐time control of photonic logic operations.

## 5 Discussion

In summary, we successfully integrated graphene into a flexible PDMS matrix to create a graphene/PDMS composite that combines optical activity with stretchability. This flexible composite exhibits a strain‐tunable spatial self‐phase modulation (SSPM) effect, where the $\chi_{\mathrm{monolayer}}^{\left(3\right)}$ decreases from 1.357 × 10^−7^ to 6.125 × 10^−8^ e.s.u. as the applied strain *ε* increases from 0% to 40%. Our investigation on the mechanism of the strain‐dependent SSPM effect reveals that strain simultaneously alters both the effective number of light‐interacting layers and the crystal structure of graphene, thereby modulating its nonlinear optical properties. Leveraging the stable SSPM response and excellent strain recovery performance of the graphene/PDMS composite, we demonstrate a strain‐tunable optical switch and reconfigurable photonic logic gates. This work provides a novel pathway for developing SSPM‐based field‐programmable optical information processing systems and opens up new possibilities for their integration into future wearable optoelectronic devices.

To the best of our knowledge, this is the first demonstration of embedding 2D materials within a flexible PDMS matrix to achieve strain‐modulated SSPM. Compared to typical Kerr media, our system trades response speed for tunability. Most systems, such as free‐space optics, metasurfaces, and on‐chip photonics, struggle to achieve tunable nonlinear optical information processing. However, the tunable nonlinearity provided by the platform based on SSPM offers unique value (e.g., allowing the “field programming” of nonlinear activation function shapes via strain, which is highly desirable for optical neural networks). Further optimizing material response and architecture can achieve faster switching and wider functional diversity.

Future research will focus on optimizing photoelectric responses and exploring alternative external field modulation methods (magnetic fields, electric fields, and temperature, etc.) to enhance the responsiveness and efficiency of tunable SSPM photonic devices. However, new challenges arise, such as applying magnetic fields that may potentially complicate device fabrication and increase costs, while temperature variations may alter the mechanical properties of PDMS, necessitating careful thermal control strategies. Beyond graphene, we also plan to investigate composites of other 2D materials integrated with PDMS, which may potentially enhance response sensitivity and extend functionality into the infrared spectrum for high‐speed and broadband information processing. We anticipate that this research will inspire further innovations in developing flexible materials and devices for optomechanical and optical communication applications.

## Funding

This work was supported by the National Natural Science Foundation of China (12504394), the Natural Science Foundation of Hebei Province (A2024203011), the Innovation Capability Improvement Project of Hebei Province (22567605H), the Hubei Provincial Science and Technology Program Project (2024EHA071), and the Beijing National Laboratory for Condensed Matter Physics (2024BNLCMPKF020).

## Conflicts of Interest

The authors declare no conflicts of interest.

## Supporting information




**Supporting File**: advs75955‐sup‐0001‐SuppMat.docx.

## Data Availability

The data that support the findings of this study are available from the corresponding author upon reasonable request.
