# Supplementary material for: Strain‐Modulated Reconfigurable Optical Information Processing in Flexible Graphene/PDMS
Source: Adv Sci (Weinh). 2026 Jun 3:e75955. Online ahead of print. doi: 10.1002/advs.75955 (PMC13336435; doi:10.1002/advs.75955)
Supplement: Supplementary file 1 — Supporting File: advs75955‐sup‐0001‐SuppMat.docx. [file ADVS-9999-e75955-s001.docx]

Supporting Information

Strain-Modulated Reconfigurable Optical Information Processing in Flexible Graphene/PDMS

Zexin Cui^a, b^, Lihua Tong^a^, Yuehua Wang^a^, Mengting Jiang^a^, Jiaxu Sun^a^, Huabo Song^a^, Qifan Li^a^, Guangyuan Cui^a^, Churui Guo^a^, Wentao Meng^a^, Shaoya Wang^a^, Yuee Chen^a,^*, Yanling Wu^a^, Xiaodan Xu^a,^*

**1. The calculation of the number of effective layers**

To quantify the thickness of graphene contributing to the nonlinear optical signal, we estimated the effective number of graphene layers (*N*_eff_) distributed within the PDMS matrix, based on the graphene concentration and sample dimensions. The composite was prepared by dispersing 1 mg of graphene powder into 10 mL of PDMS precursor, yielding a graphene concentration of c = 0.1 mg/mL. This corresponds to a carbon molar concentration of *ρ* = *c*/*M*_c_ = 8.33 × 10^-3^ mol/L, where *M*_c_ = 12 g/mol is the molar mass of carbon. The entire suspension was cured into a solid film, from which a rectangular sample of volume *V*_2_ = 2.2×10^-4^ L was excised for SSPM measurements. *V*_2_ is the physically relevant volume for determining *N*_eff_. The total number of graphene unit cells in the composite is therefore *N* = *ρ*/2 × *V*_2_ × *N*_A_ = 5.516×10^17^, *N*_A_ is the Avogadro constant, and the factor 2 accounts for the two carbon atoms per unit cell (space group *P*6/*mmm*, *a* = *b* = 2.468 Å).

The number of unit cells required to cover one effective optical interaction layer can then be estimated from the in-plane area per unit cell and the surface area of the sample:

$$\begin{aligned} m=\frac{the surface area of the sample}{unit cell area in the ab plane}=\frac{10\times20\times{10}^{-6} m^{2}}{2.468\times2.468\times\sin\left( 60^{\circ} \right)\times{10}^{-20} Å^{2}}=3.79\times{10}^{15} \end{aligned}$$

Hence, the number of effective layers of graphene/PDMS is *N*_eff_ = *N*/*m* = 146. This value corresponds to the mean number of graphene layers contributing to the SSPM signal across the illuminated region.

**2. Robustness and spatial uniformity of graphene /PDMS**

The graphene/PDMS composite exhibits excellent long-term stability and spatial uniformity. As shown in Figure S1a, the SSPM diffraction pattern remains unchanged after six months of storage under ambient conditions, demonstrating superior temporal stability. Furthermore, benefiting from the mechanical robustness of the substrate, the sample surface can be repeatedly cleaned by wiping without causing material damage. To assess spatial uniformity, SSPM diffraction was excited across different regions of the sample, yielding diffraction patterns with consistent ring numbers (Figure S1b). This confirms the highly homogeneous spatial characteristics of the composite.

**Figure S1.** a) Robustness of graphene /PDMS. b) Spatial uniformity of graphene /PDMS.

**3. The SSPM measurements in pristine PDMS**

To exclude the substrate contributions, we conducted SSPM measurements on pristine PDMS under 532 nm laser excitation (Figure S2). There is no observable SSPM effect at this wavelength, indicating that PDMS only acts as a package and has no SSPM response.

**Figure S2.** The SSPM effect of PDMS at a wavelength of 532 nm.

**4. Uniform morphology of PDMS under strain**

Under 0% – 40% strain, the PDMS morphology only underwent uniform thinning (from 1.1 to 0.96 mm), with almost no other types of deformation (as shown in Figure S3a). Furthermore, we monitored the laser intensity transmitted through pure PDMS under various strains (0%, 10%, 20%, 30%, 40%). The beam profile consistently maintained a Gaussian distribution, although the focal intensity and waist radius slightly changed (Figure S3b). To rule out this effect, we performed SSPM experiments on the released graphene/PDMS under these specific light beams. The results show that the strain-induced changes in the beam profile are insufficient to affect the number of SSPM diffraction rings, only slight differences in the profile. Therefore, the strain-tuned variation in the number of diffraction rings is mainly attributed to the intrinsic nonlinearity of graphene, rather than interference from PDMS.


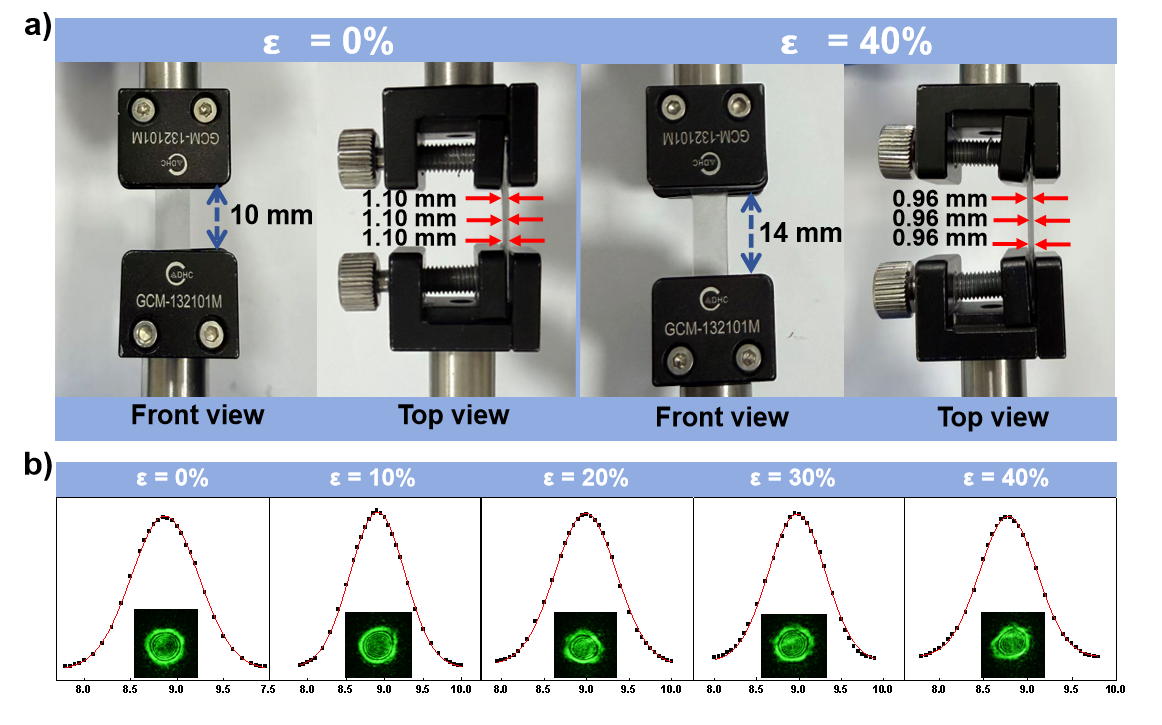


**Figure S3.** a) Morphology photos of graphene/PDMS samples under 0% and 40% strain. b) Beam profile of 532 nm laser after passing through PDMS with different strains.

**5. The mechanism of SSPM response time**

For liquid systems, “wind chime” model [1] provides a good explanation for the SSPM process. The duration needed for the emergence of SSPM diffraction ring can be represented as follow:

$$T=\frac{\varepsilon_{r}\pi\eta\xi Rc}{1.72\left( \varepsilon_{r}-1 \right)Ih}$$

PDMS, as a solid elastomer, possesses inherent viscoelastic properties. The viscoelasticities of graphene/PDMS can be measured by dynamic mechanical analyzer (DMA), as shown in Figure S4. Here, the dynamic mechanical damping factor (tan δ) is deﬁned as [2]:

$$tan \delta=\frac{G''}{G'}=\frac{viscous loss modulus}{elastic storage modulus}$$

G′ and G′′ indicate the elastic and viscous properties of the elastomer, respectively. The viscous loss modulus represents the viscosity (liquid characteristic) of PDMS.Within the frequency sweep of 1 - 10 Hz, the viscous loss modulus ranges from 0.049 to 0.097 MPa. It indicates that 2D material flakes can still be reoriented in PDMS, and the “wind chime” model remains applicable. The dynamic viscosity (*ŋ*') is related to the loss modulus (G") and frequency (ω), *ŋ*' = G"/ω [3]. The *ŋ*' = 10 KPa∙s for ω =10 Hz. We use the value *ɛ*_r_ = 3.5 for graphene, R is the radius of sample flakes, approximately ~ 2 μm. *h* is the thickness of the sample, ~ 30 nm. *I* = 56.72 W/cm^2^. *ξ* is the proportion of the package (wrapping sample sheets) excited by light to the total system, which is estimated to be closer to zero. Here, *ξ* is estimated to be 1.0×10^−9^. As a result, the value of *T* is calculated to be 0.90 s. In addition, thermal nonlinearity may exist during the interaction between light and material, which further delays the response time of the SSPM.

**Figure S4**. Dynamic mechanical test of the graphene/PDMS.

**6. Electronic band structure simulation of graphene with applied strain**

The graphene band structure was simulated at different magnitudes and angles strains, as shown in Figure S5. In the unstrained state, graphene exhibits a vanishing bandgap (*E*_g_ = 0 eV), characteristic of a semimetal. When applying uniaxial strain along the zigzag direction (0% – 8%), the bandgap increases almost linearly with the increase of tensile strain, reaching up to 0.99 eV at 8%. In contrast, fixing the strain value at 5% and rotating the direction counterclockwise from the zigzag axis (15°, 30°, 45°) will result in a decrease in the bandgap value of graphene. First-principles DFT calculations were performed with the Vienna ab initio simulation package (VASP) with the Perdew–Burke–Ernzerhof generalized gradient approximation (PBE-GGA). A monolayer graphene structure surface was prepared for a 2×2×1 unit cell with a 15 Å vacuum layer along the normal z direction. The plane-wave cutoff energy was set to 500 eV. A 12×12×1 Monkhorst–Pack k-point grid is employed in the calculations. The lattice constant of unstrained graphene structure was set to 0.242 nm. The strained graphene was obtained by applying extension on x-axes (y-axes) with a fixed value while the value of y-axes (x-axes) was tuned as the system reaches its lowest total energy. In structure relaxation, atoms were fully relaxed until the interatomic forces are less than 0.1 eV/nm.

**Figure S5.** Calculated graphene band structures under varying strain magnitudes and angles.

**7. Thickness-dependence of *n*_2_**

Graphene/PDMS were prepared with varying thicknesses ranging from 0.95 to 1.19 mm. All thickness-varied samples were fabricated with identical graphene concentration, dimensions, and ambient conditions to ensure comparability. As depicted in Figure S6, the measured *n*_2_ exhibits minimal variation, ranging from 4.91 × 10^-5^ cm^2^/W to 6.15 × 10^-5^ cm^2^/W. With strain vary from 0% to 40%, the sample thickness changes from 1.10 to 0.96 mm, resulting in an *n*_2_ variation of approximately 14% (from 5.70×10^−5^ to 4.91×10^−5^ cm^2^/W), which is far smaller than the ~ 66% reduction in *n*_2_ induced by strain tuning (from 5.743×10^−5^ to 1.962×10^−5^ cm^2^/W). Therefore, the observed strain-tunable nonlinearity primarily originates from changes in the electronic structure of graphene, rather than simply thickness variation.

**Figure S6.** *n*_2_ of graphene/PDMS with different thicknesses.

**References**

[1] Wu, Y. *et al.* Emergence of electron coherence and two-color all-optical switching in MoS_2_ based on spatial self-phase modulation. *Proc. Natl. Acad. Sci.* **2015,** 112, 11800-11805.

[2] Young O J, Kim S, Baik H K, et al. Conducting polymer dough for deformable electronics. *Adv. Mater* **2016**, 28, 4455-4461.

[3] Benbow J J, Cogswell F N, Cross M M. On the dynamic response of viscoelastic fluids. *Rheol. Acta* **1976**, 15(5), 231-237.
